# Decomposing inequality in Maternal and Child Health (MCH) services in Nepal

**DOI:** 10.1186/s12889-023-15906-2

**Published:** 2023-05-29

**Authors:** Shreezal G.C., Naveen Adhikari

**Affiliations:** Central Department of Economics, Tribhuvan University, Kirtipur, 44600 Kathmandu, Nepal

**Keywords:** ANC, Postnatal, SBA delivery, Inequality, Nepal

## Abstract

**Background:**

About 75.5% of women in Nepal’s urban areas receive at least four ANC visits, compared to 61.7% of women in the country’s rural areas. Similarly, just 34% of women in the lowest wealth quintile give birth in a medical facility compared to 90% of women in the richest group. As a result of this inequality, the poor in emerging nations suffer since those who are better off can make greater use of the healthcare than those who are less fortunate. This study aims to examine and decompose the contributions of various socioeconomic factors towards MCH service inequality in Nepal in the years 2011 and 2016.

**Methods:**

Inequality in MCH services was estimated using concentration curves and their corresponding indices using data from Nepal Demographic Health Survey (NDHS) 2011 and 2016. We examined the inequality across three MCH service outcomes: less than 4 ANC visits, no postnatal checkups within 2 months of delivery and no SBA delivery and decomposed them across observed characteristics of the mothers aged between 15 and 49. Furthermore, Oaxaca-blinder decomposition approach was used to measure and decompose the inequality differential between two time periods.

**Results:**

Inequality in MCH services was prevalent for all 3 MCH outcomes in 2011 and 2016, respectively. However, the concentration indices for <4 ANC visits, no SBA delivery, and no postnatal checkups within 2 months of birth increased from -0.2184, -0.1643, and -0.1284 to -0.1871, -0.0504, and -0.0218 correspondingly, showing the decrease in MCH services inequality over two time periods. Wealth index, women’s literacy, place of living, mother’s employment status, and problem of distance to reach nearest health facility were the main contributors.

**Conclusion:**

We find that MCH services are clearly biased towards the women with higher living standards. National policies should focus on empowering women through education and employment, along with the creation of health facilities and improved educational institutions, in order to address inequalities in living standards, women’s education levels, and the problem of distance. Leveraging these factors can reduce inequality in MCH services.

**Supplementary Information:**

The online version contains supplementary material available at 10.1186/s12889-023-15906-2.

## Background

The unequal distribution of healthcare services is a prevalent issue in low and middle-income countries, resulting in higher morbidity rates and lower health service utilization among the less affluent segments of society [1–4]. Maternal and Child Health (MCH) services, in particular, are crucial indicators of healthcare inequality, as this disparity can exist not only in low-income countries but also in some well-developed regions [5]. The provision of MCH services is essential in preventing maternal and newborn deaths, as well as promoting the well-being of future generations, ultimately leading to economic prosperity.

Nepal serves as a notable example of health inequality since its health facilities have not been able to reach the entire population properly. Around 16.67% of Nepal’s populace falls below the poverty line, with 95% of them residing in rural areas [6, 7]. Moreover, 60% of rural households require over 30 minutes to reach the nearest government health facility [8]. To address this inequity, the Nepal government has introduced health sector programs aimed at ensuring equity in health services for various socio-economic groups [9]. One such program, the Safe Motherhood program (Aama program), provides monetary incentives of Nrs. 400 to mothers who complete four antenatal care visits, covers transportation costs for mothers, and offers free institutional delivery. Health workers also receive Nrs. 300 incentives for delivering these packages [10]. The continuation of these programs has resulted in a 25% increase in antenatal care visits, a 12% increase in postnatal checkups, and a 22% increase in institutional deliveries from 2011 to 2016 [8]. Despite this progress, a significant proportion of infant and child deaths still occur in the lowest and second lowest wealth quintiles of Nepal [8]. The infant mortality rate and child mortality rate in the lowest wealth quintile are 50 and 12 per 1000 live births, respectively, compared to 20 and 4 per 1000 live births in the highest wealth quintile. Moreover, many studies have documented sluggish progress in maternal and newborn health [11, 12]. These findings have spurred researchers from various disciplines to investigate the possible reasons behind such slow progress and health inequality in Nepal.

The existing health inequality research has primarily focused on measuring health morbidity or health service utilization. The concentration index and its decomposition are commonly used methods to accurately measure health inequality. For instance, in a study of child malnutrition in Vietnam, Wagstaff et al. [13] found that the education status of parents and household consumption disproportionately affected the poor. Subsequent studies have used variations of this methodology and different proxies for child health in developing nations. They have mostly identified wealth status, immunization coverage, parental education, and access to health facilities as major contributors to inequality in child health [14–16]. Inequality in adult health is also prevalent in general and mental health, tobacco consumption, and smoking habits, with lower quintiles experiencing poorer health outcomes [1, 17, 18]. This health inequality can also be attributed to unobserved characteristics of health service consumers [19, 20]. Studies have shown that preventive health care services, essential for all, are excessively utilized by people with higher living standards than their counterparts [21, 22].

In the context of Maternal and Child Health (MCH) services, pro-rich distribution of better utilization is common in South Asian and African countries such as India, Zimbabwe, Bangladesh, and Nepal, leaving the poor at a disadvantage. The gap in health services utilization between the rich and poor in these countries is often attributed to the mother’s and partner’s literacy status, rural place of residence, and poor economic status. Several studies have investigated maternal and child health (MCH) service inequality in Nepal, with findings emphasizing the significant impact of poor economic status and socio-economic characteristics such as mother’s and partner’s literacy [23–26]. Despite the progress in MCH services in terms of utilization, such progress cannot be found in disadvantaged groups which necessitates targeted interventions [27]. In Nepal, inequalities in maternal and child health services persist, with significant disparities affecting the most vulnerable and marginalized populations. Nepal continues to face challenges in reducing maternal and child mortality rates, which remain among the highest in South Asia [28]. The most significant disparities in maternal and child health outcomes are linked to poverty, geographic location, and social status. For example, women from low-income households, rural areas, and ethnic minority groups are more likely to experience poor health outcomes and have limited access to healthcare services [23]. Recent studies also show that women utilize quality MNH services with better health facility capacity, especially the private ones since they provide better quality health services [29, 30]. While the existing studies have noted a decrease in MCH service inequality between 2011 and 2016, the drivers of such decline are unknown. Thus, this study aims to contribute to the existing literature by using the standard decomposition approach and the Oaxaca-Blinder decomposition method to analyze the inter-temporal relationship of MCH service inequality between two distinct time periods. The study’s results will help policymakers in Nepal design targeted interventions and address health inequality in line with the objectives of the Nepal Health Sector Strategy Plan [9]. By identifying the potential determinants of health inequality, this study will provide evidence-based recommendations to improve maternal and child health outcomes in Nepal.

## Methods

### Data and sample

The study utilizes the latest two rounds of data obtained from the sixth and seventh phase (2011 & 2016) of National Demographic Health Survey of Nepal [8, 31]. Data along with written consent has been obtained from the DHS program [32]. The survey has Multi-stage stratified cluster sampling design in both rounds of survey with inverse probability weights for the representation of population. However, the process of stratification in the latest round is a bit different to that of the sixth wave.

The domains in the sixth round of survey were defined as cross-sections of three ecological zones and five Nepalese development regions. Due to a small population, the cross-section of three development regions: the Western, Mid-western, and Far-western and Mountain zones have been combined into one, resulting in 13 domains. The domains are further subdivided into urban and rural areas of residence, yielding a total of 25 strata. The Enumeration Areas (EAs) were wards, with 95 in the urban and 194 in the rural regions. In the seventh round of the survey, the sample was chosen in two stages in rural areas and three stages in urban areas. This round’s strata were defined as a cross-section of provinces and place of residence, yielding 14 strata consisting of 184 EAs in urban and 199 EAs in rural areas. Since the provinces of Nepal did not exist before 2015, we incorporated the data on three ecological zones available in both the sixth and seventh rounds to control for the geographical characteristics in our analysis. DHS 2011 and DHS 2016 contain 12,674 and 12,862 married women sample aged 15 to 49, respectively. In this study, the sample is restricted to mothers who received MCH services such as Antenatal care visits, Postnatal care within two months of delivery, and Skilled Birth Attendant delivery. Details of the sample are presented in Table 1. All the analysis have been conducted in R studio version 4.2 using ‘IC2’ package from a publicly available repository [33] for concentration curves as well as their respective indices and ‘tidy verse’ package and base R functionalities for data cleaning and decomposition analysis.

**Table 1:** Summary of outcomes

Health Outcomes	2016	2011	*p*-value$^{a}$
**ANC visits**			<0.001
At least 4 ANC	2,773(69%)	2,078(50%)	
Less than 4 ANC	1,225(31%)	2,071(50%)	
Sample size (N)	3998	4148	
**Baby postnatal checkup within 2 months of delivery**			<0.001
Yes	1,492(37%)	1,933(47%)	
No	2,506(63%)	2,216(53%)	
Sample size (N)	3998	4148	
**Advised SBA delivery**			0.6
Yes	2,800(74%)	2,576(73%)	
No	962(26%)	940(27%)	
Sample size (N)	3,762	3,516	

$^{a}$ chi-squared test with Rao & Scott’s second-order correction; Wilcoxon rank-sum test for complex survey samples

#### Variables definition and measurement

The health-service outcome variables include ANC visits, postnatal checkup within 2 months of delivery and Skilled Birth Attendant (SBA) delivery to capture the different dimensions of the MCH services utilization. These standard variables are WHO identified domains of MCH services which are dichotomized in the analysis represented by “1”, else, “0”. The poor utilization of MCH services are coded as “1” for easy interpretation [24, 34] since poor services corroborate with “ill health” variables in the health-inequality literature [13]. Hence, the health outcomes in our analysis include <4 ANC visits, no postnatal checkup and no SBA delivery that represent poor utilization of MCH services.

To understand the determinants of these “ill-health” variables as well the inequalities and their respective changes, the covariates capturing different demographic and socio-economic characteristics are included. The health literature identify different predisposing and enabling factors to be instrumental in explaining the health service utilization [35]. An extensive review of literature [14, 16, 20, 23, 24, 36–48] was conducted in order to identify these factors (Supplementary Table 1). Predisposing factors constitute demographic and social characteristics such as women’s age group (15 to 24, 25 to 34, 35 to 49), sex of household head (male, female), marital status (others, married), religion (Hindu, Buddhist, Muslim, Kirat, Christian) and number of children. Enabling factors include those that allows a mother to use more of the available health services such as wealth index (poorest, poorer, middle, richer, richest), place of residence (urban, rural), ecological zones (Mountains, Hills, Terai), mother’s education (no education, primary, secondary, higher), partner’s education (uneducated, educated) and distance in reaching the nearest health facility (not a problem, problem).

### Empirical strategy

#### Measuring inequality

The concentration index is used in this study to quantify the income related inequality with respect to health variables. The concentration index used in health inequality is based on the Gini concentration index which is particularly used to measure the relative income or wealth inequality in economics [49]. However, the index can be used to measure the concentration in the distribution of any variables. The Gini index can also be expressed in terms of the Lorenz curve which plots the cumulative income or wealth against the cumulative proportion of population as per their income. The Gini index is twice the area between the line of equality (45$^{\circ }$ line) and Lorenz curve. The value of the Gini index lies between 0 and 1. Mathematically, it can be expressed in terms of definite integral as follows:

1 $$\begin{aligned} GI= & {} 2\int _0^1 [y - L(x)]dx \nonumber \\= & {} 1-2 \int _0^1 L(x)dx \nonumber \\ \end{aligned}$$

where *GI* is the Gini index, $y=x$ is the line of equality and *L*(*x*) is the lorenz curve.

The concentration curve in health reflects health inequality which uses a similar approach to the Gini Index. Under the concentration curve, the cumulative percentage of outcome health variable is plotted on y-axis instead of income or wealth variable against the cumulative percentage of population ranked by cumulative economic status on x-axis. The concentration index is defined as twice the area between the concentration curve and the line of equality (45$^{\circ }$ line). Kakwani et al. [50] have denoted concentration index in their paper as follows:

2 $$\begin{aligned} C= & {} 1-2\int _0^1 L(s)ds \nonumber \\= & {} 2cov(H_i,r_i)/\bar{H} \nonumber \\ \end{aligned}$$

where *C* is the concentration index and *L*(*s*) is the health inequality curve in (2). If the concentration curve coincides with the equality line, the value of the concentration index is 0. The index takes positive value if *L*(*s*) lies below the equality line and vice versa. The concentration index can also be represented in the covariance form where $H_i$ is the health variable, $r_i$ is the fractional rank of population according to their living standard and $\bar{H}$ is the mean of $H_i$.

The concentration curve is obtained by plotting the cumulative proportion of population as per their income or any Socio-Economic Status (SES) on x-axis and cumulative proportion of health variable y ranked by living standard on y-axis. The value of CI lies between -1 and +1. The concentration curve lies above the diagonal if it takes a negative value. In this case, the outcome variable is concentrated more on the poor or disadvantaged groups. The concentration curve lies below the diagonal when CI is positive. In such cases, the outcome studied is concentrated more on advantageous groups. If the CI is 0, the outcome is said to be equally distributed among rich and poor. In this case, the concentration curve coincides with the line of equality. Thus, unlike Lorenz curve which shows pure inequalities, concentration curve is used to analyze socio-economic inequalities in health.

The concentration index derived in Eq. (2) can also be written as follows:

3 $$\begin{aligned} C = \frac{2}{n\bar{H}} \sum \limits _{i=1}^n H_i . r_i -1 \nonumber \\ \end{aligned}$$

Where C is the concentration index, n is the number of individuals, $H_i$ is the health variable score, $\bar{H}$ is the mean of *H* and $r_i$ is the fractional rank of $i^{th}$ individuals as per their living standards. The model proposed by Wagstaff et al. [13] is used to decompose the health inequality. However, the concentration index lies between $\bar{H}-1$ and $1-\bar{H}$ in the case of binary dependent variable [51].

#### Decomposition analysis

The concentration index is used as a standard measure to assess the inequality in the health variable among the people belonging to different living standards [50]. However, the concentration index only shows if there is inequality in the health variable or not. So, the method introduced by Wagstaff et al. [13] on decomposing the health inequality is widely used in order to identify the factors of the inequality. The logistic regression model linking health variables $H_i$ to a set of predictors $x_k$ can be formulated as:

4 $$\begin{aligned} ln\left[ \frac{P_r(H_i)}{1 - P_r(H_i)}\right] = \alpha + \sum \limits _k \beta _k x_{ki} + \epsilon _i \nonumber \\ \end{aligned}$$

From Eqs. (3) and (4), the decomposed form of concentration index can be written as:

5 $$\begin{aligned} C = \sum \limits _k \frac{\beta _k \bar{x}_k }{\bar{H}}.C_k + \frac{GC_\epsilon }{\bar{H}} \nonumber \\ \end{aligned}$$

where $\beta _k$ represents average marginal effects obtained from Eq. (4) and $\frac{\beta _k\bar{x}_k }{\bar{H}}.C_k$ represents inequality explained by the covariates and $\frac{GC_\epsilon }{\bar{H}}$ represents the residual part of the inequality.

Equation (5) decomposes the health inequality to a set of *k* determinants. However, it does not measure how the inequalities have changed over time. One way to examine such changes would be to differentiate the inequalities between two time periods. However, such an approach wouldn’t allow us to capture the determinants of such changes. To overcome this issue, the study incorporates Oaxaca-Blinder decomposition approach [52, 53] in order to estimate the inequality differential between two time periods.

6 $$\begin{aligned} \triangle C = \sum \limits _k \zeta _{k_t}(C_{k_t} - C_{k_{t-1}}) + \sum \limits _k C_{kt-1}(\zeta _{k_t}- \zeta _{k_{t-1}}) + \triangle \frac{GC_{\epsilon _t}}{\bar{H}_t} \nonumber \\ \end{aligned}$$

Alternatively, Eq. (6) can also be written as follows:

7 $$\begin{aligned} \triangle C = \sum \limits _k \zeta _{k_{t-1}}(C_{k_t} - C_{k_{t-1}}) + \sum \limits _k C_{k_t}(\zeta _{k_t}- \zeta _{k_{t-1}}) + \triangle \frac{GC_{\epsilon _t}}{\bar{H}_t} \nonumber \\ \end{aligned}$$

where $\zeta _{kt}$ and $\zeta _{kt-1}$ represent elasticity of health variable *H* with respect to the covariates *x* in 2016 and 2011 respectively.

## Results

### Summary statistics

Table 1 shows the summary statistics of different MCH service outcomes for the year 2011 and 2016. The proportion of women receiving at least 4 ANC visits increased from 50% in 2011 to 69% in 2016. In the case of newborn care, 47% of the women reported that they received postnatal checkup within 2 months of delivery in 2011 which declined to 37% in 2016. The figure for advised SBA delivery increased from 73% to 74% in 2016, nevertheless, the difference was statistically insignificant. Similarly, Table 2 presents the summary statistics of socio-economic characteristics of the mothers. About 40% to 42% of the total mothers belonged to the age group of 15 to 24 and 48% to 51% belonged to the age group of 25 to 34. More than 70% of the mothers reported that the head of their households were male. About 19% to 21% of the mothers belonged to poorest wealth quintile, whereas 16% to 18% of them were richest. About 10% resided in urban areas in 2011 which increased to 56% in 2016. Most of the mothers were Hindu (84% to 86%) and the least were Kirat (1.3% to 1.6%) and Christian (1.2% to 2.1%). We can also observe that higher percentage of mothers were found to attend higher level of education in 2016 (15% to 16%) compared to 2011 (6.3% to 7.5%). Similarly, the proportion of educated partner’s were found to increase from 79% in 2011 to 86% in 2016. Whereas, the overall proportion of mothers reporting problem of distance in reaching the nearest health facility increased from 50% to 58%.

**Table 2:** Summary of socio-economic characteristics

	ANC visits			Postnatal Checkups			SBA delivery		
Characteristics	2016, N$^{a}$ = 3,998$^{a}$	2011, N$^{a}$ = 4,148$^{a}$	*p*-value$^{b}$	2016, N$^{a}$ = 3,998$^{a}$	2011, N$^{a}$ = 4,148$^{a}$	*p*-value$^{b}$	2016, N$^{a}$ = 3,762$^{a}$	2011, N$^{a}$ = 3,516$^{a}$	*p*-value$^{b}$
**Age Group**			0.001			0.001			0.3
15 to 24	1,606 (40%)	1,662 (40%)		1,606 (40%)	1,662 (40%)		1,553 (41%)	1,480 (42%)	
25 to 34	2,033 (51%)	1,980 (48%)		2,033 (51%)	1,980 (48%)		1,901 (51%)	1,709 (49%)	
35 to 49	359 (9.0%)	507 (12%)		359 (9.0%)	507 (12%)		308 (8.2%)	326 (9.3%)	
**Wealth Index**			0.5			0.5			>0.9
Poorest	822 (21%)	979 (24%)		822 (21%)	979 (24%)		712 (19%)	657 (19%)	
Poorer	839 (21%)	899 (22%)		839 (21%)	899 (22%)		782 (21%)	733 (21%)	
Middle	863 (22%)	873 (21%)		863 (22%)	873 (21%)		836 (22%)	790 (22%)	
Richer	830 (21%)	748 (18%)		830 (21%)	748 (18%)		802 (21%)	699 (20%)	
Richest	643 (16%)	649 (16%)		643 (16%)	649 (16%)		631 (17%)	637 (18%)	
**Sex of Household Head**			0.029			0.029			0.082
Male	2,796 (70%)	3,050 (74%)		2,796 (70%)	3,050 (74%)		2,629 (70%)	2,564 (73%)	
Female	1,202 (30%)	1,099 (26%)		1,202 (30%)	1,099 (26%)		1,133 (30%)	952 (27%)	
**Marital Status**			0.4			0.4			0.2
Unmarried	35 (0.9%)	45 (1.1%)		35 (0.9%)	45 (1.1%)		25 (0.7%)	34 (1.0%)	
Married	3,963 (99%)	4,104 (99%)		3,963 (99%)	4,104 (99%)		3,737 (99%)	3,482 (99%)	
**Place of Residence**			<0.001			<0.001			<0.001
Urban	2,223 (56%)	418 (10%)		2,223 (56%)	418 (10%)		2,122 (56%)	392 (11%)	
Rural	1,775 (44%)	3,730 (90%)		1,775 (44%)	3,730 (90%)		1,639 (44%)	3,124 (89%)	
**Ecological Zone**			>0.9			>0.9			0.8
Mountain	269 (6.7%)	306 (7.4%)		269 (6.7%)	306 (7.4%)		242 (6.4%)	237 (6.7%)	
Hill	1,608 (40%)	1,669 (40%)		1,608 (40%)	1,669 (40%)		1,493 (40%)	1,326 (38%)	
Terai	2,120 (53%)	2,174 (52%)		2,120 (53%)	2,174 (52%)		2,027 (54%)	1,953 (56%)	
**Religion**			<0.001			<0.001			<0.001
Hindu	3,421 (86%)	3,444 (83%)		3,421 (86%)	3,444 (83%)		3,218 (86%)	2,946 (84%)	
Buddhist	178 (4.4%)	360 (8.7%)		178 (4.4%)	360 (8.7%)		170 (4.5%)	263 (7.5%)	
Muslim	251 (6.3%)	235 (5.7%)		251 (6.3%)	235 (5.7%)		245 (6.5%)	213 (6.1%)	
Kirat	63 (1.6%)	58 (1.4%)		63 (1.6%)	58 (1.4%)		50 (1.3%)	48 (1.4%)	
Christain	85 (2.1%)	51 (1.2%)		85 (2.1%)	51 (1.2%)		79 (2.1%)	45 (1.3%)	
**Mother’s Education**			<0.001			<0.001			<0.001
No education	1,257 (31%)	1,822 (44%)		1,257 (31%)	1,822 (44%)		1,108 (29%)	1,369 (39%)	
Primary	777 (19%)	835 (20%)		777 (19%)	835 (20%)		724 (19%)	713 (20%)	
Secondary	1,345 (34%)	1,229 (30%)		1,345 (34%)	1,229 (30%)		1,313 (35%)	1,172 (33%)	
Higher	619 (15%)	263 (6.3%)		619 (15%)	263 (6.3%)		616 (16%)	262 (7.5%)	
**Partner’s Education**			<0.001			<0.001			0.003
Uneducated	542 (14%)	872 (21%)		542 (14%)	872 (21%)		475 (13%)	622 (18%)	
Educated	3,456 (86%)	3,276 (79%)		3,456 (86%)	3,276 (79%)		3,286 (87%)	2,893 (82%)	
**Occupation**			<0.001			<0.001			<0.001
Not working	1,549 (39%)	1,150 (28%)		1,549 (39%)	1,150 (28%)		1,479 (39%)	1,047 (30%)	
Working	2,449 (61%)	2,999 (72%)		2,449 (61%)	2,999 (72%)		2,283 (61%)	2,469 (70%)	
**Distance**			0.11			0.11			0.006
Not a problem	1,669 (42%)	1,902 (46%)		1,669 (42%)	1,902 (46%)		1,619 (43%)	1,763 (50%)	
Problem	2,329 (58%)	2,247 (54%)		2,329 (58%)	2,247 (54%)		2,143 (57%)	1,753 (50%)	
**Number of Children**	2.14 (1.35)	2.40 (1.58)	<0.001	2.14 (1.35)	2.40 (1.58)	<0.001	2.07 (1.29)	2.20 (1.41)	0.037

$$^{a}$$ N (%); Mean (SD)

$^{b}$ chi-squared test with Rao & Scott’s second-order correction; Wilcoxon rank-sum test for complex survey samples

### Health concentration curves and indices

**Fig. 1:**
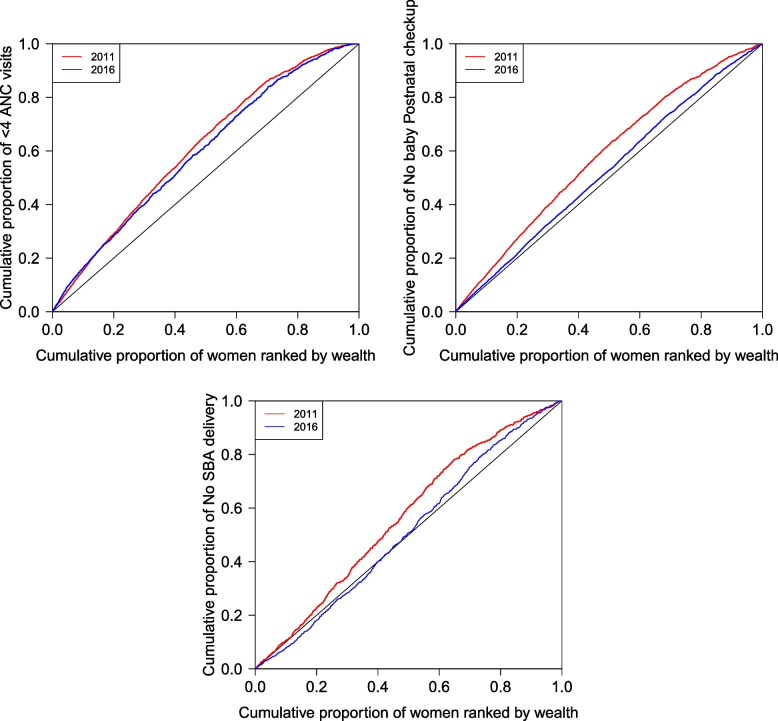
Concentration curves for $<4$ ANC visits, No postnatal checkup and No SBA delivery in 2011 and 2016

Figure 1 presents concentration curves for $<4$ ANC visits, no postnatal checkup, and no SBA delivery. All of these MCH service outcomes have inequality curves that are above the line of inequality, implying that poor utilization of MCH services is concentrated on mothers with low living standards. Despite the presence of pro-rich MCH service distribution in Nepal, the concentration curves for 2016 were slightly lower than those for 2011. This result is consistent with the negative concentration indices shown in Table 3. In 2011, the concentration indices for $<4$ ANC visits, no postnatal checkup, and no SBA delivery were -0.2184, -0.1643, and -0.1284. In 2016, these indices increased to -0.1871, -0.0504, and -0.0218 respectively, approaching the equality line.

**Table 3:** Concentration indices for health outcomes

	$<4$ ANC visits		No Postnatal checkup		No SBA Delivery	
	2011	2016	2011	2016	2011	2016
Weighted CI	-0.2184	-0.1871	-0.1643	-0.0504	-0.1284	-0.0218
Unweighted CI	-0.2258	-0.2053	-0.1646	-0.0458	-0.1133	-0.0096
Standard Error (S.E.)	0.0090	0.0132	0.0086	0.0068	0.0175	0.0162
Sample size (N)	4,148	3,998	4,148	3,998	3,516	3,762

### Odd ratios from logistic regression

Table 4 shows the factors that contribute to low utilization of MCH services in Nepal (estimation based on Eq. (4)). The results from multicollinearity and model specification tests are provided in Supplementary material Table 1 to 5. In comparison to the poorest wealth quintile, our findings show that a mother’s chances of having 4 ANC visits and no postnatal check up within 2 months of delivery decrease as she climbs up the living-standard ladder. These odds fell from 41% to 59% for women in the wealthier quintile and from 55% to 77% for those in the richest quintile. Next, compared to uneducated mothers, mothers with primary, secondary, and higher levels of education had lower odds of poor utilization of MCH services. Furthermore, as one continued up the educational level ladder, such odds were found to decrease. Similarly, having an educated partner reduced such odds by 39% across all three outcomes in 2011, but the odds were not statistically significant in 2016. The findings were similar for the problem of distance to reach the nearest health facility, where the odds were statistically not significant in 2016. In 2011, however, the odds of poor utilization of MCH services were 28% to 45% higher for mothers who experienced such problems.

**Table 4:** Odd ratios for health outcomes

	< 4ANC visits				No Postnatal Checkup				No SBA delivery			
	2011		2016		2011		2016		2011		2016	
Characteristic	OR$^{a}$	*p*-value	OR$^{a}$	*p*-value	OR$^{a}$	*p*-value	OR$^{a}$	*p*-value	OR$^{a}$	*p*-value	OR$^{a}$	*p*-value
**Age group**												
15 to 24	-		-		-		-		-		-	
25 to 34	0.78	0.013	0.76	0.008	0.86	0.15	0.96	0.7	0.88	0.3	0.81	0.038
35 to 49	0.69	0.052	0.59	0.003	0.82	0.3	0.92	0.6	0.81	0.3	0.72	0.085
**Wealth Index**												
Poorest	-		-		-		-		-		-	
Poorer	0.77	0.077	0.75	0.074	0.78	0.089	0.85	0.2	1.04	0.8	1.17	0.3
Middle	0.64	0.011	0.57	<0.001	0.58	<0.001	0.70	0.009	1.09	0.7	0.93	0.7
Richer	0.41	<0.001	0.49	<0.001	0.43	<0.001	0.59	<0.001	0.73	0.2	0.98	>0.9
Richest	0.23	<0.001	0.45	0.002	0.37	<0.001	0.42	<0.001	0.68	0.2	0.79	0.3
**Sex of Household Head**												
Male	-		-		-		-		-		-	
Female	0.86	0.082	0.86	0.088	0.93	0.5	0.85	0.042	0.96	0.8	0.86	0.13
**Marital status**												
Unmarried	-		-		-		-		-		-	
Married	1.24	0.6	0.47	0.074	1.29	0.5	1.31	0.5	5.28	0.009	0.64	0.3
**Place of residence**												
Urban	-		-		-		-		-		-	
Rural	1.09	0.6	1.27	0.072	1.11	0.4	1.04	0.7	1.15	0.4	1.19	0.3
**Ecological zone**												
Mountain	-		-		-		-		-		-	
Hill	1.24	0.2	1.04	0.9	1.51	0.026	1.38	0.14	1.60	0.046	1.24	0.4
Terai	1.41	0.070	1.41	0.2	1.57	0.021	1.75	0.015	2.88	<0.001	2.21	0.004
**Religion**												
Hindu	-		-		-		-		-		-	
Buddhist	1.36	0.039	0.91	0.7	1.72	<0.001	0.99	>0.9	1.44	0.3	1.22	0.5
Muslim	0.87	0.6	1.14	0.6	1.01	>0.9	1.07	0.7	0.99	>0.9	0.67	0.018
Kirat	1.35	0.5	1.39	0.4	1.60	0.12	0.84	0.6	1.76	0.3	1.13	0.7
Christain	1.96	0.072	1.43	0.2	1.26	0.5	0.84	0.6	0.53	0.2	1.00	>0.9
**Mother’s Education**												
No education	-		-		-		-		-		-	
Primary	0.58	<0.001	0.69	0.005	0.70	0.001	1.08	0.5	0.84	0.2	0.66	0.001
Secondary	0.41	<0.001	0.43	<0.001	0.59	<0.001	0.91	0.5	0.51	<0.001	0.60	<0.001
Higher	0.14	<0.001	0.15	<0.001	0.32	<0.001	0.72	0.031	0.40	0.004	0.44	<0.001
**Partner’s Education**												
Uneducated	-		-		-		-		-		-	
Educated	0.61	<0.001	0.75	0.015	0.61	<0.001	1.11	0.4	0.61	0.004	1.01	>0.9
**Occupation**												
Not working	-		-		-		-		-		-	
Working	0.87	0.2	0.75	0.005	1.00	>0.9	0.78	0.005	0.77	0.12	0.66	<0.001
**Distance**												
Not a problem	-		-		-		-		-		-	
Problem	1.28	0.012	1.12	0.3	1.11	0.3	0.79	0.010	1.45	0.006	1.06	0.6
**No. of children**	1.37	<0.001	1.33	<0.001	1.21	<0.001	1.15	<0.001	1.10	0.043	1.06	0.15
Sample size (N)	4,148		3,998		4,148		3,998		3,516		3,762	
AIC	4181.515		4345.18		4603.31		5223.31		3541.59		4067.59	

$^{a}$OR = Odds Ratio

### Decomposition of concentration index

The standard decomposition of the concentration index for $<4$ ANC visits is presented in Table 5. The wealth index contributed more than 50% to inequality, with the richest group alone contributing 35% in 2011 and 34% in 2016. Mother’s secondary and higher education levels were the next highest contributors, accounting for 32% and 46% of the inequality in 2011 and 2016, respectively. In fact, the inequality in higher education level contributed more to the inequality ($C_{k_t} = 0.6928; C_{k_{t-1}} = 0.4394$) in ANC visits than the elasticity ($\zeta _{k_t} = 0.0482; \zeta _{k_{t-1}} = 0.1652$). Similarly, the inequality in the problem of distance in reaching nearest health facility was found to be concentrated on the poor ($C_{k_t} = 0.2057; C_{k_{t-1}} = 0.1565$) and contributed significantly to the inequality when compared to the elasticity in 2011 and 2016 respectively. The unexplained part contributed -0.68% in 2011 and -3.09% in 2016, indicating that the covariates in our analysis explained the majority of the inequality in $<4$ ANC visits.

**Table 5:** Decomposition of CI for <4 ANC visits

Variables	Elasticities		Concentration indices		Explained contribution		Percentage contribution	
	2011	2016	2011	2016	2011	2016	2011	2016
**Age group**								
15 to 24	-	-	-	-	-	-	-	-
25 to 34	-0.0441	-0.0809	0.0547	0.0589	-0.0024	-0.0048	1.1050	2.5108
35 to 49	-0.0165	-0.0266	-0.3176	-0.1546	0.0052	0.0041	-2.3963	-2.1695
**Wealth Index**								
Poorest	-	-	-	-	-	-	-	-
Poorer	-0.0234	-0.0376	-0.3112	-0.3791	0.0073	0.0142	-3.3365	-7.5083
Middle	-0.0385	-0.0746	0.1160	0.0468	-0.0045	-0.0035	2.0449	1.8406
Richer	-0.0657	-0.0891	0.5067	0.4706	-0.0333	-0.0419	15.2324	22.1039
Richest	-0.0915	-0.0763	0.8436	0.8394	-0.0772	-0.0641	35.3361	33.7735
**Sex of Household Head**								
Male	-	-	-	-	-	-	-	-
Female	-0.0152	-0.0251	-0.0077	-0.0441	0.0001	0.0011	-0.0535	-0.5842
**Marital Status**								
Others	-	-	-	-	-	-	-	-
Married	0.0810	-0.4651	0.0014	0.0008	0.0001	-0.0004	-0.0519	0.1961
**Place of residence**								
Urban	-	-	-	-	-	-	-	-
Rural	0.0280	0.0613	-0.0645	-0.1963	-0.0018	-0.0120	0.8271	6.3435
**Ecological zones**								
Mountains	-	-	-	-	-	-	-	-
Hills	0.0321	0.0093	-0.1760	-0.1308	-0.0057	-0.0012	2.5883	0.6430
Terai	0.0679	0.1039	0.1948	0.1657	0.0132	0.0172	-6.0568	-9.0736
**Religion**								
Hindu	-	-	-	-	-	-	-	-
Buddhist	0.0100	-0.0023	-0.1081	0.0084	-0.0011	0.0000	0.4952	0.0100
Muslim	-0.0030	0.0048	0.0931	0.2085	-0.0003	0.0010	0.1281	-0.5242
Kirat	0.0016	0.0032	-0.1261	-0.0951	-0.0002	-0.0003	0.0902	0.1580
Christian	0.0031	0.0046	0.1537	-0.0087	0.0005	0.0000	-0.2153	0.0209
**Mother’s Education**								
No education	-	-	-	-	-	-	-	-
Primary	-0.0465	-0.0503	-0.0889	-0.1795	0.0041	0.0090	-1.8934	-4.7599
Secondary	-0.1122	-0.1841	0.3285	0.0807	-0.0368	-0.0149	16.8695	7.8305
Higher	-0.0482	-0.1652	0.6928	0.4394	-0.0334	-0.0726	15.3055	38.2689
**Partner’s education**								
Uneducated	-	-	-	-	-	-	-	-
Educated	-0.1511	-0.1482	0.0893	0.0418	-0.0135	-0.0062	6.1763	3.2646
**Occupation**								
Not working	-	-	-	-	-	-	-	-
Working	-0.0368	-0.1032	-0.1265	-0.1401	0.0047	0.0145	-2.1313	-7.6217
**Distance**								
Not a problem	-	-	-	-	-	-	-	-
Problem	0.0507	0.0386	-0.2057	-0.1565	-0.0104	-0.0060	4.7762	3.1843
**No. of children**	0.2848	0.3547	-0.1215	-0.0812	-0.0346	-0.0288	15.8432	15.1843
**Explained Contributions**					-0.2199	-0.1956	100.6828	103.0917
**Residual**					0.0015	0.0059	-0.6828	-3.0917
**CI**					-0.2184	-0.1897	100	100

Table 6 shows the concentration index decomposition for no postnatal checkup. Overall, we find that the richer (19.41% in 2011; 35.83% in 2016) and richest (33.51% in 2011; 83.69% in 2016) wealth quintiles contributed significantly to postnatal checkup inequality. Mother’s education level, particularly secondary and higher education, was the next major contributor, accounting for approximately 25.68% in 2011 and 17.89% in 2016. Similar to the results in Table 5, the magnitudes of secondary and higher level education concentration indices are much higher than their respective elasticities, indicating that inequality in education level contributed to the pro-rich distribution of health services. The unexplained contributions were 1.13% in 2011 and 6.7% in 2016. Table 7 shows the decomposition of the concentration index for no SBA delivery. Similarly to the previous findings, we find that the mother’s education level and higher wealth quintiles are the most important determinants of inequality in SBA delivery. In 2011, the richest and richest wealth quintiles contributed 13.76% and 28.78% of the inequality, respectively. The contribution of the inequality in higher education level itself was very high ($C_{k_t} = 0.6577$) compared to its elasticity ($\zeta _{k_t} = 0.0427$). In 2016, the wealth index and education level were found to have very high ($> 100\%$) contributions to inequality. The contributions of those determinants, however, are largely offset by the Terai zone and mothers’ occupation status. Overall, we find that the wealth index and mother’s education level are the two most significant contributors to MCH services inequality across all three outcomes.

**Table 6:** Decomposition of CI for no postnatal checkup

Variables	Elasticities		Concentration indices		Explained Contribution		Percentage Contribution	
	2011	2016	2011	2016	2011	2016	2011	2016
**Age group**								
15 to 24	-	-	-	-	-	-	-	-
25 to 34	-0.0292	-0.0066	0.0554	0.0596	-0.0016	-0.0004	0.9853	0.7771
35 to 49	-0.0094	-0.0028	-0.3179	-0.1541	0.0030	0.0004	-1.8091	-0.8541
**Wealth Index**								
Poorest	-	-	-	-	-	-	-	-
Poorer	-0.0215	-0.0123	-0.3108	-0.3787	0.0067	0.0046	-4.0620	-9.2123
Middle	-0.0471	-0.0269	0.1155	0.0482	-0.0054	-0.0013	3.3105	2.5768
Richer	-0.0630	-0.0382	0.5062	0.4723	-0.0319	-0.0181	19.4075	35.8287
Richest	-0.0652	-0.0504	0.8446	0.8364	-0.0551	-0.0422	33.5147	83.6958
**Sex of Household Head**								
Male	-	-	-	-	-	-	-	-
Female	-0.0076	-0.0179	-0.0075	-0.0432	0.0001	0.0008	-0.0346	-1.5335
**Marital Status**								
Others	-	-	-	-	-	-	-	-
Married	0.0985	0.0993	0.0014	0.0008	0.0001	0.0001	-0.0840	-0.1576
**Place of residence**								
Urban	-	-	-	-	-	-	-	-
Rural	0.0384	0.0059	-0.0645	-0.1959	-0.0025	-0.0012	1.5069	2.3119
**Ecological zones**								
Mountains	-	-	-	-	-	-	-	-
Hills	0.0657	0.0492	-0.176	-0.1321	-0.0116	-0.0065	7.0341	12.9009
Terai	0.0931	0.1089	0.1949	0.1667	0.0181	0.0181	-11.0467	-36.0087
**Religion**								
Hindu	-	-	-	-	-	-	-	-
Buddhist	0.0184	-0.0001	-0.1071	0.0092	-0.0020	0.0000	1.1999	0.0021
Muslim	0.0003	0.0015	0.0926	0.2103	0.0000	0.0003	-0.0143	-0.6302
Kirat	0.0026	-0.0010	-0.1267	-0.0943	-0.0003	0.0001	0.1981	-0.1927
Christian	0.0011	-0.0013	0.1535	-0.0078	0.0002	0.0000	-0.1056	-0.0208
**Mother’s Education**								
No education	-	-	-	-	-	-	-	-
Primary	-0.0298	0.0050	-0.0892	-0.1789	0.0027	-0.0009	-1.6198	1.7823
Secondary	-0.0659	-0.0109	0.3281	0.0791	-0.0216	-0.0009	13.1501	1.7096
Higher	-0.0297	-0.0185	0.6935	0.4413	-0.0206	-0.0082	12.5361	16.1830
**Partner’s Education**								
Uneducated	-	-	-	-	-	-	-	-
Educated	-0.1545	0.0328	0.0892	0.0417	-0.0138	0.0014	8.3895	-2.7152
**Occupation**								
Not working	-	-	-	-	-	-	-	-
Working	-0.0003	-0.0534	-0.1265	-0.1406	0.0000	0.0075	-0.0208	-14.8842
**Distance**								
Not a problem	-	-	-	-	-	-	-	-
Problem	0.0221	-0.0484	-0.2056	-0.1572	-0.0045	0.0076	2.7663	-15.0967
**No. of children**	0.1852	0.1044	-0.1212	-0.0813	-0.0225	-0.0085	13.6654	16.8412
**Explained Contributions**					-0.1624	-0.0470	98.8676	93.3035
**Residual**					-0.0019	-0.0034	1.1324	6.6965
**CI**					-0.1643	-0.0504	100	100

**Table 7:** Decomposition of CI for No SBA delivery

Variables	Elasticities		Concentration indices		Explained contribution		Percentage contribution	
	2011	2016	2011	2016	2011	2016	2011	2016
**Age group**								
15 to 24	-	-	-	-	-	-	-	-
25 to 34	-0.0395	-0.0767	0.0615	0.0623	-0.0024	-0.0048	1.89	21.9
35 to 49	-0.0124	-0.0182	-0.278	-0.1198	0.0034	0.0022	-2.68	-10
**Wealth Index**								
Poorest	-	-	-	-	-	-	-	-
Poorer	0.0053	0.024	-0.4179	-0.4139	-0.0022	-0.0099	1.71	45.5
Middle	0.0138	-0.0109	0.0155	0.0162	0.0002	-0.0002	-0.17	0.81
Richer	-0.0402	-0.0035	0.4391	0.4518	-0.0177	-0.0016	13.76	7.34
Richest	-0.0451	-0.0262	0.8191	0.8327	-0.037	-0.0218	28.78	100
**Sex of Household Head**								
Male	-	-	-	-	-	-	-	-
Female	-0.0073	-0.0325	-0.0224	-0.0395	0.0002	0.0013	-0.13	-5.9
**Marital Status**								
Others	-	-	-	-	-	-	-	-
Married	0.7262	-0.3439	0.0012	0.0007	0.0009	-0.0002	-0.68	1.1
**Place of residence**								
Urban	-	-	-	-	-	-	-	-
Rural	0.0778	0.0523	-0.0701	-0.197	-0.0055	-0.0103	4.25	47.3
**Ecological zones**								
Mountains	-	-	-	-	-	-	-	-
Hills	0.0916	0.0484	-0.1381	-0.1251	-0.0126	-0.0061	9.85	27.8
Terai	0.3492	0.2846	0.15	0.1544	0.0524	0.0439	-40.8	-202
**Religion**								
Hindu	-	-	-	-	-	-	-	-
Buddhist	0.0187	0.0065	-0.031	0.0058	-0.0006	0	0.45	-0.2
Muslim	-0.0003	-0.0165	-0.0114	0.1871	0	-0.0031	0	14.2
Kirat	0.0055	0.0012	-0.1838	-0.1189	-0.001	-0.0001	0.79	0.66
Christian	-0.0046	0	0.1496	-0.0441	-0.0007	0	0.53	0
**Mother’s Education**								
No education	-	-	-	-	-	-	-	-
Primary	-0.0262	-0.0601	-0.1279	-0.1829	0.0033	0.011	-2.61	-50
Secondary	-0.1496	-0.1332	0.2843	0.0655	-0.0425	-0.0087	33.13	40
Higher	-0.0427	-0.0936	0.6577	0.424	-0.0281	-0.0397	21.87	182
**Partner’s Education**								
Uneducated	-	-	-	-	-	-	-	-
Educated	-0.2863	0.0059	0.0757	0.0375	-0.0217	0.0002	16.88	-1
**Occupation**								
Not working	-	-	-	-	-	-	-	-
Working	-0.1244	-0.1814	-0.1278	-0.1416	0.0159	0.0257	-12.4	-118
**Distance**								
Not a problem	-	-	-	-	-	-	-	-
Problem	0.122	0.0245	-0.2113	-0.1618	-0.0258	-0.004	20.08	18.2
**No. of children**	0.1431	0.0804	-0.098	-0.0727	-0.014	-0.0058	10.92	26.8
**Explained contributions**					-0.1354	-0.032	105.42	146.51
**Residual**					0.007	0.0102	-5.42	-46.51
**CI**					-0.1284	-0.0218	100	100

### Oaxaca-blinder decomposition

**Fig. 2:**
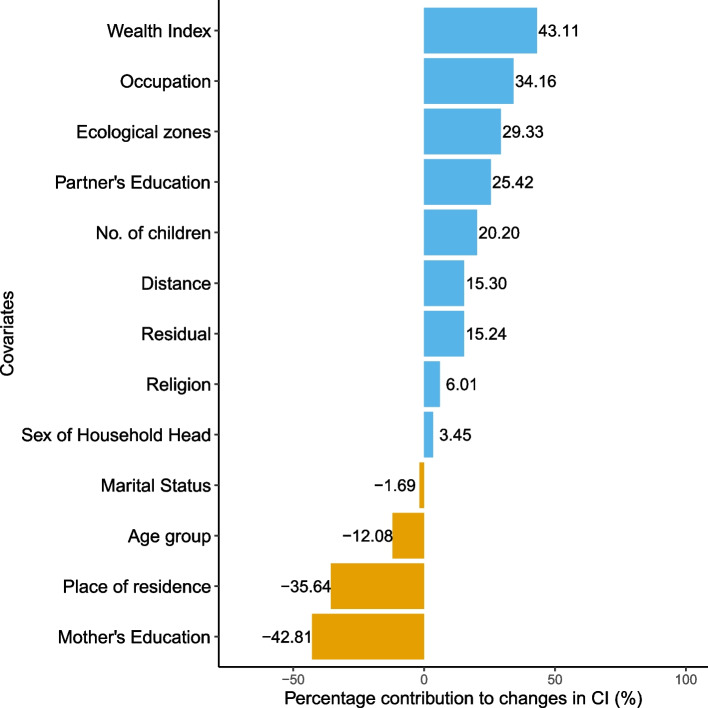
Oaxaca decomposition for <4 ANC visits

Table 8 presents the decomposition analysis for the changes in inequality in $<4$ ANC visits between 2011 and 2016. The overall difference in the concentration index between these two periods was found to be 0.0287 of which 85.76% of it was attributable to the differences in the observed characteristics and the remaining 15.24% due to the changes in the residuals. While mother’s higher education level (-136.48%) contributed to the increase in inequality, secondary level and primary level of education offset such magnitude by 17.05% and 76.61% respectively. Overall, mother’s education contributed about 42.81% to worsening the inequality in $<4$ ANC visits (Fig. 2). Similarly, place of residence (35.64%), age group (12.08%) and marital status (1.69%) also contributed in worsening the inequality. Whereas, wealth index (43.11%) was highest contributor in reducing the inequality in 2016 followed by occupation status (34.16%), ecological zones (29.33%), partner’s education (25.42%), number of children (20.20%), distance (15.30%), religion (6.01%) and sex of the household head (3.45%).

**Table 8:** Oaxaca blinder decomposition for $<4$ ANC visits

Variables	Variation (1)		Variation (2)		Total	
	$\zeta _{k_t}(C_{k_t} - C_{k_{t-1}})$	$C_{kt-1}(\zeta _{k_t}- \zeta _{k_{t-1}})$	$\zeta _{k_{t-1}}(C_{k_t} - C_{k_{t-1}})$	$C_{k_t}(\zeta _{k_t}- \zeta _{k_{t-1}})$	Total	%
**Age group**						
15 to 24						
25 to 34	-0.0003	-0.0020	-0.0002	-0.0022	-0.0023	-8.19
35 to 49	-0.0043	0.0032	-0.0027	0.0016	-0.0011	-3.90
**Wealth Index**						
Poorest	-	-	-	-	-	-
Poorer	0.0026	0.0044	0.0016	0.0054	0.0070	24.24
Middle	0.0052	-0.0042	0.0027	-0.0017	0.0010	3.39
Richer	0.0032	-0.0119	0.0024	-0.0110	-0.0087	-30.19
Richest	0.0003	0.0128	0.0004	0.0127	0.0131	45.66
**Sex of Household Head**						
Male	-	-	-	-	-	-
Female	0.0009	0.0001	0.0006	0.0004	0.0010	3.45
**Marital Status**						
Others	-	-	-	-	-	-
Married	0.0003	-0.0008	0.0000	-0.0004	-0.0005	-1.69
**Place of residence**						
Urban	-	-	-	-	-	-
Rural	-0.0081	-0.0021	-0.0037	-0.0065	-0.0102	-35.64
**Ecological zones**						
Mountains	-	-	-	-	-	-
Hills	0.0004	0.0040	0.0015	0.0030	0.0044	15.45
Terai	-0.0030	0.0070	-0.0020	0.0060	0.0040	13.88
**Religion**						
Hindu	-	-	-	-	-	-
Buddhist	-0.0003	0.0013	0.0012	-0.0001	0.0011	3.70
Muslim	0.0006	0.0007	-0.0003	0.0016	0.0013	4.44
Kirat	0.0001	-0.0002	0.0000	-0.0002	-0.0001	-0.36
Christian	-0.0007	0.0002	-0.0005	0.0000	-0.0005	-1.78
**Mother’s Education**						
No education	-	-	-	-	-	-
Primary	0.0046	0.0003	0.0042	0.0007	0.0049	17.05
Secondary	0.0456	-0.0236	0.0278	-0.0058	0.0220	76.61
Higher	0.0419	-0.0810	0.0122	-0.0514	-0.0392	-136.48
**Partner’s Education**						
Uneducated	-	-	-	-	-	-
Educated	0.0070	0.0003	0.0072	0.0001	0.0073	25.42
**Occupation**						
Not working	-	-	-	-	-	-
Working	0.0014	0.0084	0.0005	0.0093	0.0098	34.16
**Distance**						
Not a problem	-	-	-	-	-	-
Problem	0.0019	0.0025	0.0025	0.0019	0.0044	15.30
**No. of children**	0.0143	-0.0085	0.0115	-0.0057	0.0058	20.20
**Explained contributions**					0.0243	84.76
**Residual**					0.0044	15.24
**Total**					0.0287	100.00

**Fig. 3:**
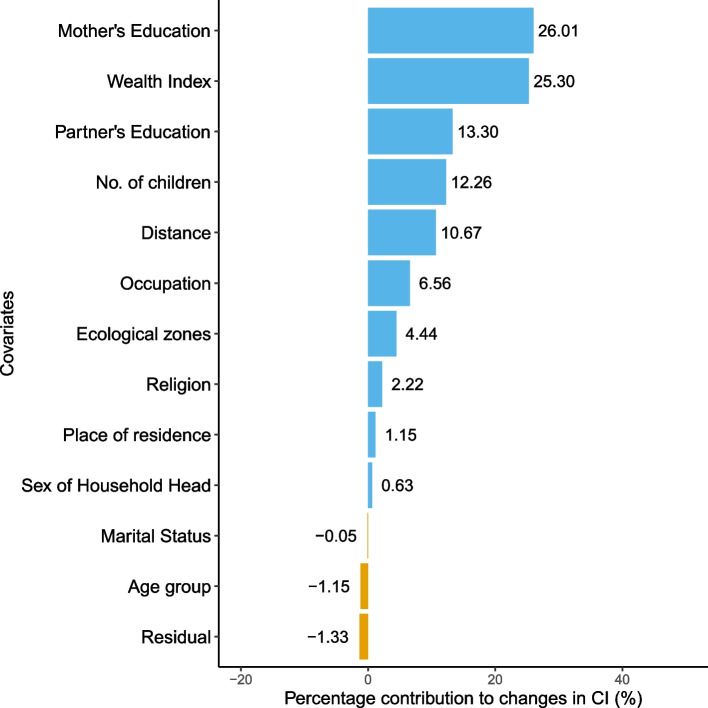
Oaxaca decomposition for No baby postnatal checkup within 2 months of delivery

**Fig. 4:**
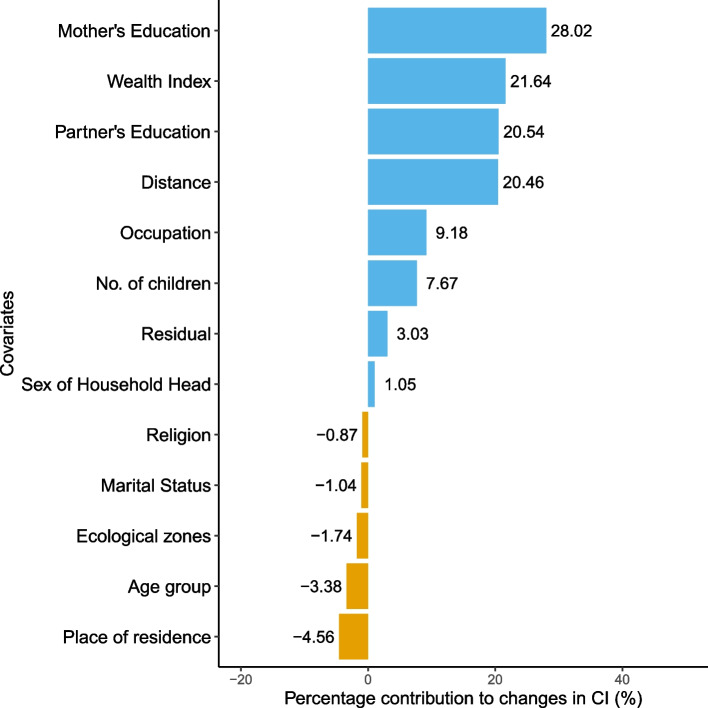
Oaxaca decomposition for No SBA delivery

The inequality gap in postnatal checkup between the year 2011 and 2016 was found to be wider than that of $<4$ ANC visits where 101.33% of the changes were explained by observable factors and -1.33% was the residual as shown in Table 9. As shown in Fig. 3, a majority of variables contributed to the reduction of inequality, including mother’s education (26.01%), wealth index (25.30%), partner’s education (13.30%), number of children (12.26%), distance (10.67%), occupation (6.56%), ecological zones (4.44%), religion (2.22%), place of residence (1.15%) and sex of household head (0.63%). However, the only observable factors that worsened inequality in 2016 were marital status (0.05%) and age group (1.15%). Similarly, Table 10 shows the Oaxaca Blinder decomposition results for no SBA delivery. The difference in concentration index between 2011 and 2016 was 0.1066 out of which 96.97% was explained by the covariates. Mother’s education was the largest contributor in inequality reduction followed by wealth index, partner’s education, number of children, distance, occupation, ecological zones, religion, place of residence and sex of household head as presented in Fig. 4.

**Table 9:** Oaxaca blinder decomposition analysis for no postnatal checkup

Variables	Variation (1)		Variation (2)		Total	
	$\zeta _{k_t}(C_{k_t} - C_{k_{t-1}})$	$C_{kt-1}(\zeta _{k_t}- \zeta _{k_{t-1}})$	$\zeta _{k_{t-1}}(C_{k_t} - C_{k_{t-1}})$	$C_{k_t}(\zeta _{k_t}- \zeta _{k_{t-1}})$	Total	%
**Age group**						
15 to 24	-	-	-	-		
25 to 34	0.0000	0.0013	-0.0001	0.0013	0.0012	1.08
35 to 49	-0.0005	-0.0021	-0.0015	-0.0010	-0.0025	-2.23
**Wealth Index**						
Poorest	-	-	-	-	-	-
Poorer	0.0008	-0.0029	0.0015	-0.0035	-0.0020	-1.78
Middle	0.0018	0.0023	0.0032	0.0010	0.0041	3.64
Richer	0.0013	0.0125	0.0021	0.0117	0.0138	12.14
Richest	0.0004	0.0125	0.0005	0.0123	0.0129	11.31
**Sex of Household Head**						
Male	-	-	-	-	-	-
Female	0.0006	0.0001	0.0003	0.0004	0.0007	0.63
**Marital Status**						
Others	-	-	-	-	-	-
Married	-0.0001	0.0000	-0.0001	0.0000	-0.0001	-0.05
**Place of residence**						
Urban	-	-	-	-	-	-
Rural	-0.0008	0.0021	-0.0050	0.0064	0.0013	1.15
**Ecological zones**						
Mountains	-	-	-	-	-	-
Hills	0.0022	0.0029	0.0029	0.0022	0.0051	4.44
Terai	-0.0031	0.0031	-0.0026	0.0026	0.0000	0.00
Religion						
Hindu	-	-	-	-	-	-
Buddhist	0.0000	0.0020	0.0021	-0.0002	0.0020	1.73
Muslim	0.0002	0.0001	0.0000	0.0003	0.0003	0.26
Kirat	0.0000	0.0005	0.0001	0.0003	0.0004	0.37
Christian	0.0002	-0.0004	-0.0002	0.0000	-0.0002	-0.14
**Women’s Education**						
No education	-	-	-	-	-	-
Primary	-0.0005	-0.0031	0.0027	-0.0062	-0.0036	-3.13
Secondary	0.0027	0.0180	0.0164	0.0043	0.0207	18.21
Higher	0.0047	0.0078	0.0075	0.0050	0.0124	10.92
**Partner’s Education**						
Uneducated	-	-	-	-	-	-
Educated	-0.0016	0.0167	0.0073	0.0078	0.0152	13.30
**Occupation**						
Not working	-	-	-	-	-	-
Working	0.0008	0.0067	0.0000	0.0075	0.0075	6.56
**Distance**						
Not a problem	-	-	-	-	-	-
Problem	-0.0023	0.0145	0.0011	0.0111	0.0122	10.67
**No. of children**	0.0042	0.0098	0.0074	0.0066	0.0140	12.26
**Explained contributions**					0.1154	101.33
**Residual**					-0.0015	-1.33
**Total**					0.1139	100.00

**Table 10:** Oaxaca blinder decomposition analysis for no SBA delivery

Variables	Variation (1)		Variation (2)		Total	
	$\zeta _{k_t}(C_{k_t} - C_{k_{t-1}})$	$C_{kt-1}(\zeta _{k_t}- \zeta _{k_{t-1}})$	$\zeta _{k_{t-1}}(C_{k_t} - C_{k_{t-1}})$	$C_{k_t}(\zeta _{k_t}- \zeta _{k_{t-1}})$	Total	%
**Age group**						
15 to 24	-	-	-	-	-	-
25 to 34	-0.0001	-0.0023	0	-0.0023	-0.0024	-2.21
35 to 49	-0.0029	0.0016	-0.002	0.0007	-0.0013	-1.18
**Wealth Index**						
Poorest	-	-	-	-	-	-
Poorer	0.0001	-0.0078	0	-0.0078	-0.0077	-7.25
Middle	0	-0.0004	0	-0.0004	-0.0004	-0.36
Richer	0	0.0161	-0.0005	0.0166	0.0161	15.07
Richest	-0.0004	0.0155	-0.0006	0.0157	0.0151	14.18
**Sex of Household Head**						
Male	-	-	-	-	-	-
Female	0.0006	0.0006	0.0001	0.001	0.0011	1.05
**Marital Status**						
Others	-	-	-	-	-	-
Married	0.0002	-0.0013	-0.0004	-0.0007	-0.0011	-1.04
**Place of residence**						
Urban	-	-	-	-	-	-
Rural	-0.0066	0.0018	-0.0099	0.005	-0.0049	-4.55
**Ecological zones**						
Mountains	-	-	-	-	-	-
Hills	0.0006	0.006	0.0012	0.0054	0.0066	6.17
Terai	0.0013	-0.0097	0.0015	-0.01	-0.0084	-7.91
**Religion**						
Hindu	-	-	-	-	-	-
Buddhist	0.0002	0.0004	0.0007	-0.0001	0.0006	0.58
Muslim	-0.0033	0.0002	-0.0001	-0.003	-0.0031	-2.9
Kirat	0.0001	0.0008	0.0004	0.0005	0.0009	0.81
Christian	0	0.0007	0.0009	-0.0002	0.0007	0.63
**Mother’s Education**						
No education	-	-	-	-	-	-
Primary	0.0033	0.0043	0.0014	0.0062	0.0076	7.16
Secondary	0.0291	0.0047	0.0327	0.0011	0.0338	31.72
Higher	0.0219	-0.0335	0.01	-0.0216	-0.0116	-10.86
**Partner’s Education**						
Uneducated	-	-	-	-	-	-
Educated	-0.0002	0.0221	0.0109	0.011	0.0219	20.54
**Occupation**						
Not working	-	-	-	-	-	-
Working	0.0025	0.0073	0.0017	0.0081	0.0098	9.18
**Distance**						
Not a problem	-	-	-	-	-	-
Problem	0.0012	0.0206	0.006	0.0158	0.0218	20.45
No. of children	0.002	0.0061	0.0036	0.0046	0.0082	7.67
**Explained contributions**					0.1034	96.97
**Residual**					0.0032	3.03
**Total**					0.1066	100

## Discussion

Our study shows declining trend of inequality in MCH services. Nevertheless, inequality still persists and puts poor group at disadvantage in terms of utilization of health services. The standard decomposition of concentration index indicated higher living standards, higher education level of mothers and problem of distance in reaching health facilities to contribute in widening the inequality gap. The Oaxaca Blinder decomposition results pointed out wealth index, partner’s education, distance and mother’s occupation status to be major contributing factors in reducing the inequality in MCH services. The findings of our study are similar to the recent studies in Nepal which identify wealth index and maternal education to be highest contributors in widening the gap in child’s nutritional status and institutional delivery between poor and non-poor [25, 54]. The decreasing trend of inequality in MCH services but the prevailing concentration of poor utilization of such services on the mothers with low standard of living can also be found in different studies conducted in Nepal [24, 26].

Overall, our results are supportive in terms of reducing inequality in MCH services in Nepal. The National Programs by GoN are consistently increasing the number of Skilled Birth Attendants (SBA) so as to facilitate birth preparedness. The monetary schemes for both mothers and health workers have been effective in narrowing down the inequality. The health systems are still transitioning ever since Nepal introduced federalism under which the municipalities are given the leadership in providing health services. Such structure has allowed local governments to establish birthing and delivery centers in villages that have facilitated institutional delivery in the rural parts [55]. Despite significant improvement in accessibility to different health centers and facilities in Nepal, the accessibility for households with longer distance from the district centers are still low compared to those in the zonal centers [56]. More autonomy should be given to the municipalities and local bodies in establishing and maintaining local health posts, clinics and hospitals such that health service seekers can have easy access to those places. Establishing hospitals at strategic locations along with better referral mechanism in local health centers can be effective in tackling the difficulty in reaching health facilities. It is also necessary to ensure proper availability of equipment and trained health workers in such local facilities. Health system readiness capacity of the government and facilities are equally important along with accessibility [57].

A major shortcoming can be seen in the demand-side policy in terms of slow progress in maternal health. The existing Safe Motherhood Programs have been successful in inducing the demand for better utilization of MCH services. However, the enabling factors such as standard of living and education level of mothers play key role in ensuring better utilization of health services. The inequality in the wealth index at richer and richest wealth quintile alone contributed more than 50% to the inequality in MCH services in our study. Therefore, it is imperative to reduce living standard related inequalities in Nepal so that a massive chunk of inequality in MCH services can be reduced. Privileged women in terms of living standards, education, access to financial institutions and other demographic characteristics are often found to utilize optimal quality Maternal and Newborn Health (MNH) services compared to their counterparts despite high coverage of such services [58].

Another significant policy lever includes mother’s education since higher education was found to be concentrated on mothers belonging to higher wealth quintiles. The inequality in mother’s education is even higher as one goes for secondary and higher levels of education in Nepal. Although the literacy rate in Nepal has improved over the years, the students still continue to drop out, especially girls. Moreover, women face pressure from their respective families to get married at the age where they should be attaining higher education. The risk of attrition from education is even higher for girls once they start with their lower secondary education and peaks at seventh or eighth grade because of early marriage [59]. The girls in Nepal are even more disadvantaged due to socially conservative attitudes towards women which is well-reflected in terms of education level [60]. Moreover, our findings are also similar to a study conducted in Nepal which identified that women with no formal education and those falling in lower wealth quintiles were the most disadvantaged groups in receiving the proper maternal care [20]. A large proportion of mothers have also reported not to be aware of free abortion services in Nepal [61]. In Nepal’s context, it is also necessary to educate other family members including husband and in-laws since mothers themselves have less autonomy. The existing birth preparedness policies should further facilitate counselling services and awareness campaigns targeting the husbands and other family members. Introducing such interventions in corroboration with existing demand side programs shall prove to be effective in intercepting the slow progress.

The results from our study should however be interpreted cautiously. Our analysis identifies the determinants of inequality and distributes it into different observable factors. Our analysis is purely based on the observational dataset due to which mothers could have self-selected themselves in using the health services. Despite such limitation, the contribution of unobserved factors to the inequality is comparatively lower to other significant determinants. In addition, the advantage lies in the use of a nationally representative DHS data set with adequate sample size which allows us to minimize such bias and generalize the result in the context of Nepal. If so, then how relevant is this study for policy implications in Nepal? Our study identifies the socio-economic groups where MCH services inequality is prevalent to a greater extent. The results from this study pre-informs target groups to implement health sector intervention programs. Policy interventions targeting mothers with low level of education and low-living standards as well as those who are unemployed and face problem of distance in reaching health facility can be instrumental in narrowing down the inequality to a much larger extent.

## Conclusions

In Nepal, the distribution and use of health services has historically been pro-rich. Health policies alone are not adequate in tackling the health-related inequality and slow progress in MNH health in Nepal since the inequality in different socio-economic characteristics contribute to the disparity in health service utilization. Despite the decreasing trend of health inequality, it is necessary to tackle the income inequality as well as inequalities in the health service determinants which were found to have major contribution in creating gap between rich and poor in terms of MCH services utilization. First, narrowing the gap in living standards of mothers is essential in addition to the current safe motherhood programs and health system strengthening programs that have been facilitating easy access to better utilization of health services. Next, policies that ensure equitable access to higher education for every mother as well as awareness campaigns catering her family members can be an effective measure to narrow down the gap in health service utilization. Interventions that empower women and mothers can help reduce the inequality in enabling factors such as education and occupation which can further have transitive effect in narrowing down the health-inequality gap in Nepal.

## Supplementary Information

**Additional file 1.**

## Data Availability

Publicly available data were used that are accessible from the DHS Program website (https://www.dhsprogram.com/data/available-datasets.cfm) upon request.
