# Correction: Decomposing inequality in Maternal and Child Health (MCH) services in Nepal

**DOI:** 10.1186/s12889-023-16087-8

**Published:** 2023-06-14

**Authors:** Shreezal G.C., Naveen Adhikari

**Affiliations:** Central Department of Economics, Tribhuvan University, Kirtipur, 44600 Kathmandu Nepal

**Correction: BMC Public Health 23, 995 (2023)**

**https://doi.org/10.1186/s12889-023-15906-2**

The original publication of this article [1] contained a typo on page 4. The incorrect and correct information is listed in this correction article, the original article has been updated.

Incorrect

The concentration curve lies below the diagonal when CI is negative

Correct

The concentration curve lies below the diagonal when CI is positive
