# Supplementary material for: Decomposing inequality in Maternal and Child Health (MCH) services in Nepal
Source: BMC Public Health. 2023 May 29;23:995. doi: 10.1186/s12889-023-15906-2 (PMC10226207; doi:10.1186/s12889-023-15906-2)
Supplement: Supplementary file 1 — Additional file 1. [file 12889_2023_15906_MOESM1_ESM.docx]

**Supplementary information**

**Decomposing inequality in Maternal and Child Health (MCH) Services in Nepal**

**Derivation of equation (3)**

$$C=\frac{2cov\left( H_{i}, r_{i} \right)}{\bar{H}}$$

$=\frac{2}{\bar{H}}[E\left( H_{i}.r_{i} \right)-E\left( H_{i} \right). E\left( r_{i} \right)]$

$=\frac{2}{\bar{H}}[\sum_{i=1}^{n} H_{i}.\frac{R_{i}}{n}-1/2 \sum_{i=1}^{n} \frac{H_{i}}{n}]$

$=\frac{2}{n\bar{H}}\sum_{i=1}^{n} H_{i}.R_{i}-1$

**Derivation of equation (5)**

$C=\frac{2}{n\bar{H}}\sum_{i=1}^{n} [\alpha+ \sum_{k} \beta_{k}x_{ki}+\epsilon_{i}].r_{i}-1$

$= \frac{2}{n\bar{H}}\left[ \alpha\sum r_{i}+\beta_{k}\sum_{i=1}^{n} x_{ki}r_{i}+ \sum r_{i}. \epsilon_{i} \right]-1$

$= \frac{2}{n\bar{H}}\left[ \alpha.\frac{n}{2}+\beta_{k}\sum_{k} \frac{C_{k}+1}{2}.n\bar{x}_{k}+GC_{\epsilon}.\frac{n}{2} \right]$ ∵ $C_{k}=\frac{2}{n.\bar{x}_{k}}\sum_{i=1}^{n} x_{i}.r_{i}-1$

$=\frac{1}{\bar{H}}\left[ \alpha+\beta_{k}\sum_{k} \left( C_{k}+1 \right)\bar{x}_{k}+GC_{\epsilon} \right]-1$

$=\frac{1}{\bar{H}}\left[ \alpha+\beta_{k}\bar{x}_{k}. \sum_{k} C_{k}+\beta_{k}\bar{x}_{k}+GC_{\epsilon} \right]-1$

= $\frac{1}{\bar{H}}\left[ \bar{H}+ \sum_{k} B_{k}\bar{x}_{k}. C_{k}+GC_{\epsilon} \right]-1$ ∵$\mu=\alpha+ \beta_{k}\bar{x}_{k}$

$=1+\sum_{k} \frac{\beta_{k}\bar{x}_{k}}{\bar{H}}. C_{k}+\frac{GC_{\epsilon}}{\bar{H}}-1$

$=\sum_{k} \frac{\beta_{k}\bar{x}_{k}}{\bar{H}}. C_{k}+\frac{GC_{\epsilon}}{\bar{H}}$

**Supplementary table 1:** List of variables identified from literature and available in Nepal Demographic Health Survey 2011 and 2016 dataset

| SN | Variable | Measurement | References |
| --- | --- | --- | --- |
| 1 | ANC Visits | Categorized as per WHO standards: at least 4 visits (0); less than 4 visits (1) | 5-6 |
| 2 | Postnatal checkup | Categorized as: Yes (0); No (1) | 7-9 |
| 3 | Advised SBA delivery | Categorized as: Yes (0); No (1) | 7-9 |
| 4 | Age Group | Categorized as: 15 to 24 (0), 25 to 34 (1) and 35 to 49 (2) | 21-22 |
| 5 | Wealth Index | Categorized as: poorest (0); poorer (1); middle (2); richer (3); richest (4) | 11-12, 18-21 |
| 6 | Sex of Household Head | Categorized as: male (0); female (1) | 15-18 |
| 7 | Marital Status | Categorized as: others (0); married (1) | 9-10, 20 |
| 8 | Place of Residence | Categorized as: urban (0); rural (1) | 10-11, 18 |
| 9 | Ecological Zone | Categorized as: mountain (0); hill (1); terai (2) | 13 |
| 10 | Religion | Categorized as: Hindu (0); Buddhist (1); Muslim (2); Kirat (3); Christian (4) | 19-20, 22 |
| 11 | Mother’s Education | Categorized as: No education (0); primary (1); secondary (2); higher (3) | 12-14, 18-21 |
| 12 | Partner’s Education | Categorized as: Uneducated (0); Educated (1) | 7 |
| 13 | Occupation Status | Categorized as: Not working (0); working (1) | 19 |
| 14 | Problem of Distance (nearest health facility) | Categorized as: Not a problem (0); problem (1) | 11 |
| 15 | Number of living children | Continuous variable | 14 |

**Supplementary table 2**: Generalized Variance Inflation Factors (GVIFs) of the variables included in <4 ANC model

|  |  | 2011 | | 2016 | |
| --- | --- | --- | --- | --- | --- |
| Variables | $df$ | $GVIF$ | ${GVIF}^{1/2df}$ | $GVIF$ | ${GVIF}^{1/2df}$ |
| Age group | 2 | 1.7968 | 1.1578 | 1.7962 | 1.1577 |
| Wealth Index | 4 | 2.063 | 1.0947 | 2.1788 | 1.1022 |
| Sex of HH | 1 | 1.0206 | 1.0102 | 1.0277 | 1.0138 |
| Marital Status | 1 | 1.0219 | 1.0109 | 1.0111 | 1.0055 |
| Place of residence | 1 | 1.1676 | 1.0806 | 1.0942 | 1.046 |
| Ecological zones | 2 | 1.5388 | 1.1138 | 1.8242 | 1.1622 |
| Religion | 4 | 1.2388 | 1.0271 | 1.2338 | 1.0266 |
| Women’s education | 3 | 1.5793 | 1.0791 | 1.6553 | 1.0876 |
| Partner’s literacy | 1 | 1.1997 | 1.0953 | 1.1906 | 1.0911 |
| Occupation | 1 | 1.3016 | 1.1409 | 1.2012 | 1.096 |
| Distance | 1 | 1.1188 | 1.0577 | 1.0936 | 1.0458 |
| No. of children | 1 | 1.8481 | 1.3594 | 1.8285 | 1.3522 |

**Supplementary table 3**: Generalized Variance Inflation Factors (GVIFs) of the variables included in no postnatal checkup model

|  |  | 2011 | | 2016 | |
| --- | --- | --- | --- | --- | --- |
| Variables | $df$ | $GVIF$ | ${GVIF}^{1/2df}$ | $GVIF$ | ${GVIF}^{1/2df}$ |
| Age group | 2 | 1.8325 | 1.1635 | 1.6855 | 1.1394 |
| Wealth Index | 4 | 2.2976 | 1.1096 | 2.2858 | 1.1089 |
| Sex of HH | 1 | 1.0208 | 1.0103 | 1.026 | 1.0129 |
| Marital Status | 1 | 1.0202 | 1.01 | 1.0083 | 1.0041 |
| Place of residence | 1 | 1.1947 | 1.093 | 1.1735 | 1.0833 |
| Ecological zones | 2 | 1.5107 | 1.1087 | 1.6062 | 1.1258 |
| Religion | 4 | 1.2234 | 1.0255 | 1.2144 | 1.0246 |
| Women’s education | 3 | 1.8144 | 1.1044 | 2.0273 | 1.125 |
| Partner’s literacy | 1 | 1.2375 | 1.1124 | 1.2352 | 1.1114 |
| Occupation | 1 | 1.2775 | 1.1303 | 1.1763 | 1.0846 |
| Distance | 1 | 1.1654 | 1.0795 | 1.1879 | 1.0899 |
| No. of children | 1 | 1.9504 | 1.3966 | 1.8355 | 1.3548 |

**Supplementary table 4**: Generalized Variance Inflation Factors (GVIFs) of the variables included in no SBA delivery model

|  |  | 2011 | | 2016 | |
| --- | --- | --- | --- | --- | --- |
| Variables | $df$ | $GVIF$ | ${GVIF}^{1/2df}$ | $GVIF$ | ${GVIF}^{1/2df}$ |
| Age group | 2 | 1.7802 | 1.1551 | 1.6872 | 1.1397 |
| Wealth Index | 4 | 2.3553 | 1.113 | 2.2034 | 1.1038 |
| Sex of HH | 1 | 1.0221 | 1.011 | 1.0225 | 1.0112 |
| Marital Status | 1 | 1.0148 | 1.0074 | 1.0076 | 1.0038 |
| Place of residence | 1 | 1.1547 | 1.0746 | 1.1252 | 1.0608 |
| Ecological zones | 2 | 1.4871 | 1.1043 | 1.6404 | 1.1317 |
| Religion | 4 | 1.2793 | 1.0313 | 1.2213 | 1.0253 |
| Women’s education | 3 | 1.8446 | 1.1074 | 1.9353 | 1.1163 |
| Partner’s literacy | 1 | 1.2763 | 1.1297 | 1.2229 | 1.1058 |
| Occupation | 1 | 1.2777 | 1.1304 | 1.1728 | 1.083 |
| Distance | 1 | 1.1291 | 1.0626 | 1.1295 | 1.0628 |
| No. of children | 1 | 1.9183 | 1.385 | 1.8319 | 1.3535 |

**Supplementary table 5: Model specification test for logistic regressions using blr_linktest command in R studio**

| ANC (2011) | Estimate | Std. Error | Z value | Pr(>\|Z\|) |
| --- | --- | --- | --- | --- |
| (Intercept) | -0.0608 | 0.0441 | -1.381 | 0.167 |
| fit | 0.9412 | 0.0350 | 26.866 | <2e-16*** |
| fit2 | -0.0212 | 0.0244 | -0.871 | 0.384 |

| ANC (2016) | Estimate | Std. Error | Z value | Pr(>\|Z\|) |
| --- | --- | --- | --- | --- |
| (Intercept) | -0.0498 | 0.0469 | -1.061 | 0.2888 |
| fit | 0.8288 | 0.0710 | 11.667 | <2e-16*** |
| fit2 | -0.0646 | 0.0358 | -1.804 | 0.0712 |

| Postnatal checkup (2011) | Estimate | Std. Error | Z value | Pr(>\|Z\|) |
| --- | --- | --- | --- | --- |
| (Intercept) | -0.0481 | 0.0426 | -1.130 | 0.259 |
| fit | 0.9016 | 0.0428 | 21.086 | <2e-16*** |
| fit2 | -0.0061 | 0.0411 | 0.149 | 0.882 |

| Postnatal checkup (2016) | Estimate | Std. Error | Z value | Pr(>\|Z\|) |
| --- | --- | --- | --- | --- |
| (Intercept) | 0.1259 | 0.0549 | 2.294 | 0.0218** |
| fit | 0.7983 | 0.1638 | 4.872 | 1.11e-0.6*** |
| fit2 | 0.0411 | 0.1501 | 0.274 | 0.7843 |

| SBA Delivery (2011) | Estimate | Std. Error | Z value | Pr(>\|Z\|) |
| --- | --- | --- | --- | --- |
| (Intercept) | -0.1508 | 0.0859 | -1.757 | 0.0789* |
| fit | 1.1680 | 0.1459 | 8.003 | 1.22e-15 *** |
| fit2 | 0.1271 | 0.0594 | 2.138 | 0.0325** |

| SBA Delivery (2016) | Estimate | Std. Error | Z value | Pr(>\|Z\|) |
| --- | --- | --- | --- | --- |
| (Intercept) | 0.0489 | 0.1281 | 0.382 | 0.703 |
| fit | 1.2623 | 0.2497 | 5.054 | 4.32e-07 *** |
| fit2 | 0.1131 | 0.1123 | 1.007 | 0.314 |

## References

1. Fox, J. and Monette, G. (1992) Generalized collinearity diagnostics, JASA 87, 178–183. <https://doi.org/10.2307/2290467>
2. Pregibon, D. 1979. Data analytic methods for generalized linear models. PhD diss., University of Toronto.
3. Pregibon, D. 1980. Goodness of link tests for generalized linear models.
4. Tukey, J. W. 1949. One degree of freedom for non-additivity.
5. Shibre, G., Mekonnen, W. Socio-economic inequalities in ANC attendance among mothers who gave birth in the past 12 months in Debre Brehan town and surrounding rural areas, North East Ethiopia: a community-based survey. *Reprod Health* **16**, 99 (2019). <https://doi.org/10.1186/s12978-019-0768-8>
6. Rahman, A., Nisha, M.K., Begum, T. *et al.* Trends, determinants and inequities of 4^+^ ANC utilisation in Bangladesh. *J Health Popul Nutr* **36**, 2 (2017). https://doi.org/10.1186/s41043-016-0078-5
7. Goli, S., Nawal, D., Rammohan, A., Sekher, T. V., & Singh, D. (2018). Decomposing the socioeconomic inequality in utilization of maternal health care services in selected countries of South Asia and sub-Saharan Africa. *Journal of biosocial science*, *50*(6), 749-769.
8. [Nawal, D.](https://www.emerald.com/insight/search?q=Dipty%20Nawal) and [Goli, S.](https://www.emerald.com/insight/search?q=Srinivas%20Goli) (2013), "Inequalities in utilization of maternal health care services in Nepal", [*Ethnicity and Inequalities in Health and Social Care*](https://www.emerald.com/insight/publication/issn/1757-0980), Vol. 6 No. 1, pp. 3-15. https://doi.org/10.1108/EIHSC-11-2012-0015
9. Goli, S., Singh, L., Jain, K. *et al.* Socioeconomic Determinants of Health Inequalities Among the Older Population in India: A Decomposition Analysis. *J Cross Cult Gerontol* **29**, 353–369 (2014). https://doi.org/10.1007/s10823-014-9251-8
10. Omotoso, K. O., & Koch, S. F. (2018). Assessing changes in social determinants of health inequalities in South Africa: A decomposition analysis. *International Journal for Equity in Health*, *17*(1), 1-13.
11. Mahumud, R. A., Alam, K., Renzaho, A. M., Sarker, A. R., Sultana, M., Sheikh, N., ... & Gow, J. (2019). Changes in inequality of childhood morbidity in Bangladesh 1993-2014: a decomposition analysis. *PloS one*, *14*(6), e0218515.
12. Memirie, S. T., Verguet, S., Norheim, O. F., Levin, C., & Johansson, K. A. (2016). Inequalities in utilization of maternal and child health services in Ethiopia: the role of primary health care. *BMC health services research*, *16*, 1-8.
13. Mehata, S., Paudel, Y. R., Dariang, M., Aryal, K. K., Lal, B. K., Khanal, M. N., & Thomas, D. (2017). Trends and inequalities in use of maternal health care services in Nepal: strategy in the search for improvements. *BioMed research international*, *2017*.
14. Bhatta, D. N., & Aryal, U. R. (2015). Paternal factors and inequity associated with access to maternal health care service utilization in Nepal: a community based cross-sectional study. *PLoS One*, *10*(6), e0130380.
15. Mulenga, J. N., Bwalya, B. B., & Chishimba, K. K. (2017). Determinants and inequalities in access to improved water sources and sanitation among the Zambian households.
16. Kavosi, Z., Rashidian, A., Pourreza, A., Majdzadeh, R., Pourmalek, F., Hosseinpour, A. R., ... & Arab, M. (2012). Inequality in household catastrophic health care expenditure in a low-income society of Iran. *Health policy and planning*, *27*(7), 613-623.
17. Kien, V.D., Van Minh, H., Giang, K.B. *et al.* Socioeconomic inequalities in catastrophic health expenditure and impoverishment associated with non-communicable diseases in urban Hanoi, Vietnam. *Int J Equity Health* **15**, 169 (2016). <https://doi.org/10.1186/s12939-016-0460-3>
18. Paredes, K. P. P. (2016). Inequality in the use of maternal and child health services in the Philippines: do pro-poor health policies result in more equitable use of services?. *International Journal for Equity in Health*, *15*(1), 1-11.
19. Jalloh, M. B., Bah, A. J., James, P. B., Sevalie, S., Hann, K., & Shmueli, A. (2019). Impact of the free healthcare initiative on wealth-related inequity in the utilization of maternal & child health services in Sierra Leone. *BMC health services research*, *19*(1), 1-15.
20. Krishnamoorthy, Y., Majella, M. G., & Rajaa, S. (2020). Equity in coverage of maternal and newborn care in India: evidence from a nationally representative survey. *Health Policy and Planning*, *35*(5), 616-623.
21. Nguhiu, P. K., Barasa, E. W., & Chuma, J. (2017). Determining the effective coverage of maternal and child health services in Kenya, using demographic and health survey data sets: tracking progress towards universal health coverage. *Tropical Medicine & International Health*, *22*(4), 442-453.
22. Oludamilola Adeyanju, Sandy Tubeuf, Tim Ensor, Socio-economic inequalities in access to maternal and child healthcare in Nigeria: changes over time and decomposition analysis, *Health Policy and Planning*, Volume 32, Issue 8, October 2017, Pages 1111–1118, <https://doi.org/10.1093/heapol/czx049>
